# Factors associated with reported modern contraceptive use among married men in Afghanistan

**DOI:** 10.1186/s12978-020-0908-1

**Published:** 2020-05-12

**Authors:** Catherine A. Packer, Sayed Haroon Rastagar, Mario Chen, Alissa Bernholc, Shafiqullah Hemat, Sediq Seddiqi, Ross McIntosh, Elizabeth Costenbader, Catherine S. Todd

**Affiliations:** 1 Global Health, Population, and Nutrition, FHI 360, 359 Blackwell Street, Suite 200, Durham, NC 27701 US; 2 FHI 360, HEMAYAT Project, Street 15, Wazir Akbar Khan, Kabul, Afghanistan; 3 Department of Health Promotion, Ministry of Public Health, Islamic Republic of Afghanistan, Masoud Circle, Wazir Akbar Khan, Kabul, Afghanistan; 4 Assess, Transform, & Reach (ATR) Consulting, Taimani, Kabul, Afghanistan

**Keywords:** Afghanistan, Men, Contraceptive use, Internally displaced persons (IDP), Gender, Decision-making, Intimate partner violence, Male engagement in family planning, Reproductive health

## Abstract

**Background:**

Afghanistan has high maternal and infant mortality which is in part driven by high fertility and low modern contraceptive use. Using modern contraceptive methods can reduce maternal and infant mortality, however there are several barriers to modern contraceptive use in Afghanistan. Married men have the potential to hinder or facilitate their wives’ contraceptive use. Internally displaced persons (IDP), a growing population in Afghanistan, are rarely included in reproductive health research. We explored whether married men’s, including IDPs’, gender-related attitudes and other factors were associated with reported modern contraceptive use to inform programming to meet reproductive health needs of married couples.

**Methods:**

Cross-sectional study using data from 885 married men determined to have contraceptive need in seven Afghan provinces. We explored associations between sociodemographic factors, IDP status, wives’ involvement in household decision-making and men’s attitudes towards intimate partner violence (IPV) with reported modern contraceptive use using logistic regression analysis.

**Results:**

Most men (78%) had ≥2 children, 60% reported any formal education, and 30% reported being IDPs. Only 38% of married men and 24% of IDPs with contraceptive need reported using modern contraception with their wives. Most (80% overall, 63% of IDPs) reported their wives’ involvement in some/all household decisions, while 47% overall and 57% of IDPs reported IPV was justified in one or more listed circumstances. In bivariate analysis, men responding that IPV was not justified in any listed circumstance were more likely and IDPs less likely to report modern contraceptive use. In multivariable analysis, involvement by wives in household decision-making (AOR 2.57; 95% CI: 1.51, 4.37), owning a radio and/or television (AOR 1.69; 95% CI: 1.10, 2.59), having more children, age, and province of interview were independently associated with reported modern contraceptive use, while IDP status was not.

**Conclusions:**

Our findings reflect positive associations between wives’ participation in household decisions and mass media exposure (television/radio ownership) with reported modern contraceptive use. Reproductive health initiatives engaging men to promote communication within couples and through mass media channels may further increase modern contraceptive use and advance Afghanistan’s family planning goals. As fewer IDPs owned a radio/television, additional outreach methods should be tested for this group.

## Plain English summary

Married men have the potential to hinder or facilitate their wives’ contraceptive use. Internally displaced persons (IDPs) are rarely included in reproductive health research though they may have different needs. We explored whether married men’s, including IDPs’, gender-related attitudes and other factors were associated with reported modern contraceptive use in a sample of married men from seven provinces in Afghanistan, where contraceptive prevalence remains low. We used cross-sectional data from a larger formative study about reproductive, maternal, newborn and child health. We included 885 married men determined to have contraceptive need from 1658 male respondents. We explored associations between sociodemographic factors, IDP status, wives’ involvement in household decision-making and men’s attitudes towards intimate partner violence (IPV) with reported modern contraceptive use using logistic regression analysis.

Men responding that IPV was not justified in any listed circumstance were more likely and IDPs less likely to report modern contraceptive use in bivariate analysis. However, in multivariable analysis, IPV attitudes and IDP status were not associated with modern contraceptive use. Greater involvement by wives in decision-making, having more children, owning a radio and/or television, age, and province of interview were independently associated with reported modern contraceptive use in multivariable analysis.

Our findings reflect positive associations between wives’ participation in household decisions and mass media exposure with reported modern contraceptive use. Reproductive health initiatives engaging men, including IDPs, to promote communication within couples and through mass media and other channels may further increase modern contraceptive use and advance Afghanistan’s FP goals.

## Background

Despite experiencing decades of conflict and ongoing insecurity, Afghanistan has made substantial progress towards improving reproductive, maternal, newborn and child health (RMNCH) since 2002 [1, 2]. However, persistent high total fertility (5.3 children) and low (20%) contraceptive prevalence rates (CPR) contribute to the highest maternal and infant mortality rates in the region [1, 3, 4]. Modern contraceptive use in Afghanistan is substantially lower than other countries in the Central and South Asian regions [5], and has not measurably changed over the past decade [2].

Family planning (FP) is among the most effective and cost-efficient strategies to reduce maternal and infant mortality and thereby improve the health of families [6, 7]. However, 25% of married women in Afghanistan have an unmet need for FP [3]. In 2016, the Government of Afghanistan made a FP2020 commitment to achieve a CPR of 30% with modern methods and to reduce unmet need by 10% by 2020 and has made some progress [8]. However, structural barriers exist to FP use in Afghanistan including poor health system infrastructure, an insufficient number of female health providers, poverty, low educational attainment, and reduced access to services due to geographic and security limitations [2, 9–11]. Additionally, individual and community level barriers, including misconceptions, limited knowledge, and negative attitudes about contraception, such as fear of side effects, beliefs that FP is counter to Islamic teachings, and norms surrounding women’s autonomy in health decision-making and mobility, also hinder FP uptake and use [2, 10–12].

Men are often important gatekeepers for women’s access to and use of modern contraception, as men are traditionally the primary household decision-makers in Afghanistan, including about their wives’ healthcare [3, 10, 13] and contraceptive use [11, 14]. Cultural practices requiring women to be accompanied by a male chaperone to leave the house restrict women’s agency and mobility to seek health services [10]. Of the few studies that have examined Afghan men’s perspectives regarding FP or actual use of FP, findings note that men endorse FP use but have misconceptions about some or all FP methods related to perceived religious prohibitions and negative impacts on future fertility [9, 12, 15–17]. Two studies evaluating interventions which engaged men (mostly via counseling couples on the safety and benefits of contraception) to increase FP use in Afghanistan found that male involvement was critical in facilitating actual contraceptive use [9, 16]. Thus, engaging men as supportive partners may help address the barriers to FP use and thereby increase modern contraceptive use to improve the health of women, infants and families in Afghanistan.

The present study included internally displaced men as a group of particular interest. Afghanistan is facing high and increasing internal displacement resulting from ongoing conflict and insecurity, and natural disasters (e.g. droughts) [18, 19]. Internally displaced persons (IDPs) often have higher levels of unemployment, poverty and lack of access to services [18], including reproductive health services [20]. Most reproductive health research does not include IDPs, though this group may have reproductive health attitudes, behaviors and needs that differ from the general population. Given the understudied nature and lack of documentation of IDPs in reproductive health research, we highlight the characteristics and needs of IDP respondents in this paper.

Understanding what characteristics are associated with married men’s reported modern contraceptive use can inform public health programs aiming to meet the reproductive health needs of Afghan couples. We conducted an analysis using data from a larger formative mixed methods study about FP and reproductive, maternal, newborn and child health (FP/RMNCH) knowledge, attitudes, and practices and media use and preferences for FP/RMNCH content among men and young people in Afghanistan [17]. The objective of this analysis was to explore what factors were associated with reported modern contraceptive use among married men in this sample. Due to men’s influence on contraceptive use in their marital union, we were interested in exploring whether men’s gender-related attitudes and behaviors were associated with reported contraceptive use. Specifically, we explored whether men’s reported involvement of their wife in household decision-making [21–28] and attitudes towards intimate partner violence (IPV) [23, 26–28] were associated with modern contraceptive use in Afghanistan as has been found, primarily in studies with women, in other settings. We also aimed to explore IDPs’ reported attitudes and contraceptive use as a group of interest. These results will inform activity design and adaptation for male engagement programs to promote FP discussion and use among married couples, including IDPs.

## Methods

### Data

Data for this analysis were collected for a cross-sectional mixed methods formative assessment conducted collaboratively by the Health Promotions Department of the Ministry of Public Health (MoPH), the United States Agency for International Development (USAID)-funded Helping Mothers & Children Thrive in Afghanistan (HEMAYAT) project, and Assess, Transform, and Reach (ATR) Consulting [17]. This parent study measured FP/RMNCH knowledge and attitudes, health-seeking behaviors, media exposure overall and to health topics, and preferred sources for FP/RMNCH messaging among male and youth target audiences to inform targeted demand generation programs for FP/RMNCH services in Afghanistan. Between March and July 2017, the parent study enrolled 1658 adult males, and 1200 school-based and 452 internally displaced male and female youth aged 15-to-25 years in urban and rural locations in seven provinces with sampled districts listed (Balkh: Mazar-i-Sharif city, Bamyan: Bamyan and Yawkawlang districts, Herat: Herat city; Zenda Jan and Injil districts, Kabul: Kabul city, Kandahar: Kandahar city and Daman District, Nangarhar: Jalalabad city; Behsud, Kama, and Khiwa districts and Takhar: Farkar, Rustaq, and Taloquan districts) in Afghanistan. The parent study was reviewed and approved by the Institutional Review Board of the MoPH in Afghanistan (355310) and the FHI 360 Protection of Human Subjects Committee (844213).

The parent study collected data from a convenience sample of adult males aged 18–49 years purposively recruited from different provinces and different groups of men. Men were recruited from predominantly male organizations (Afghan National Security Forces (ANSF) training sites, community *shuras*, and farmers collective offices) and areas with known large IDP populations. IDPs were included to ensure representation of this growing group in Afghanistan. Recruitment areas in each province were selected based on presence and accessibility of target groups in sufficient numbers and security considerations. For organizations with multiple locations within one province, recruitment sites were randomly selected prior to field activities. After obtaining appropriate government and organizational permissions, trained male staff presented a study overview to men gathered in meeting rooms within the respective organizations. Men were then counted, potential participants and alternates randomly selected, and selected individuals offered participation. In areas with large IDP populations, we engaged local community leaders, presented the study, and obtained permissions to recruit within the community. We then chose a central land-mark and used a random walk technique [29] to select a household for participant recruitment. At each selected household, one eligible male resident interested in participation was offered enrollment after confirming displaced status, until the sample size was reached. For all groups, male staff conducted verbal informed consent and administered the questionnaire in Dari or Pashto in a private room.

Typically, men are not asked about FP or other RMNCH issues in household surveys in Afghanistan, with these questions instead directed to married women of reproductive age. Questions regarding reproductive health decisions and about a man’s wife are culturally sensitive and must be approached carefully. To address this, male interviewers were trained to establish rapport with male participants by initiating “ice-breaker” conversations prior to questionnaire administration, asking less controversial questions earlier in the questionnaire, and emphasizing the anonymous nature of the survey prior to asking sensitive questions (e.g. about FP use). IDP status is also usually not queried in household surveys in Afghanistan.

**Fig. 1:**
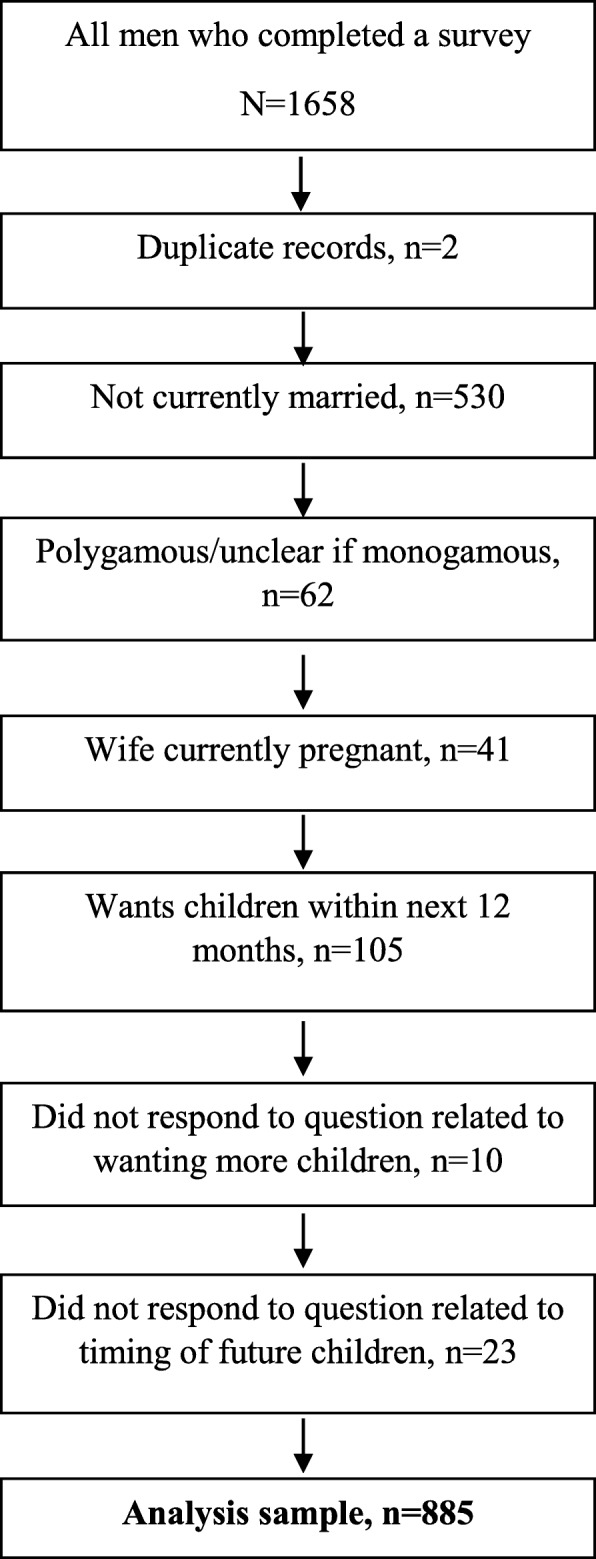
Participants excluded from analysis sample, in order of removal

For this analysis, we only included data from monogamous married men with contraceptive need, which we defined as men reporting not wanting any (more) children or wanting (more) children but not for at least 12 months (Fig. 1). We also included men who were undecided if or when they wanted (more) children. Polygamy is legal in Afghanistan, and the survey asked questions about “your wife” but did not ask men to specify which wife in cases where more than one wife was reported. Therefore, we excluded men who reported having more than one wife or men for whom it was unclear whether they had more than one wife. We also excluded men whose wives were currently pregnant, those who wanted children within the next 12 months, and those who did not respond to questions regarding whether and when they wanted more children. Of 1658 adult males completing the survey in the parent study, 773 respondents were excluded (mostly unmarried men), retaining 885 men in this analysis. We report the characteristics of the IDP respondents separately as a group of interest in our analysis.

### Measures

The primary outcome for this analysis, reported modern contraceptive use, is defined as reporting current use with wife of at least one of the following methods to prevent pregnancy: intrauterine device (IUD), implant, oral contraceptives, injectable contraceptives, male condoms, female or male sterilization, emergency contraception and lactational amenorrhea (LAM) [3]. Men were asked “Are you and your wife using any method to delay or avoid pregnancy at this time?”. Men who responded affirmatively to this question were coded as “using FP”; these men were then asked what method(s) they were using. We created a dichotomous variable for analysis for using versus not using at least one modern contraceptive method. Men who reported using withdrawal or the rhythm/standard days method only were coded as not using modern contraception.

For analysis, we selected demographic characteristics including age, number of living children, having any formal education, reported IDP status and socioeconomic characteristics including owning a motorized vehicle (household wealth and mobility) and owning a radio and/or television (household wealth and potential mass media exposure), as these could influence contraceptive use. We included province of interview and rural or urban residence due to association with reported contraceptive use [3] and, for provinces, to account for potential geographic variation in the dependent and independent factors of interest. We were also interested in exploring whether men’s gender-related attitudes and reported behaviors were associated with contraceptive use. Specifically, we assessed how men reported involving their wives in three types of household decisions and men’s attitudes towards IPV in five scenarios. We assessed both sets of questions related to these topics using Cronbach’s alpha and considered 0.70 as an acceptable level of intercorrelation to create composite variables [30], one for wives’ involvement in household decision-making and another for IPV attitudes. The household decision-making questions were: “Who usually decides how the money you earn will be used?”, “Who usually makes decisions about making major household purchases?” and, “Who usually makes decisions about health care for your family?”. We created a dichotomous variable for each question and confirmed intercorrelation (Cronbach’s alpha = 0.84) prior to combining into one categorical variable: wife not involved in any household decisions, wife involved in some decisions, and wife involved in all decisions. The IPV attitudes questions were used in the Afghanistan Demographic and Health Survey (DHS) and other international household surveys [3, 31] and query whether a husband is justified in hitting or beating his wife in five specific circumstances: if she goes out of the house without telling him; if she neglects the children; if she argues with him; if she burns the food; and if she refuses to have sex with him. We created dichotomous variables for each question (agree vs disagree). Respondents who refused to answer a question or responded “Don’t know” were set to missing for that question. After confirming intercorrelation (Cronbach’s alpha = 0.73), we created a single dichotomous variable with agreement with at least one statement that a husband is justified in beating his wife versus disagreeing with all statements.

### Analysis

We conducted descriptive analyses of participants’ sociodemographic and reproductive characteristics. We then explored associations between variables of interest and modern contraceptive use, first using bivariate logistic regression analyses to test for crude associations and second using multivariable logistic regression to examine adjusted associations. We also examined whether the associations between men’s gender-related attitudes and behaviors (i.e. IPV attitudes and household decision-making) and modern contraceptive use were different or modified by IDP status by including interaction terms in the multivariable model. We planned to include these interaction terms in the final model if they were significant. We assessed collinearity in the resulting model using tolerance and condition index statistics. We decided that variables with tolerance < 0.10 or condition indexes > 30 were to be further examined and removed from the model if necessary. Statistical significance was assessed at the 5% level. Data were analyzed using SAS (Enterprise Guide 7.1, SAS Institute Inc.).

## Results

Almost all respondents in this analysis were born in Afghanistan, most lived in rural areas and nearly half had no formal education (Table 1). Nearly three-quarters owned a radio and/or television and less than half owned a motorized vehicle. Most respondents had two or more children. Almost one-third of the respondents reported that they were IDPs and were interviewed in three of the seven provinces. Almost half of these IDPs lived in urban areas, about two-thirds owned  a radio/television and about one-third owned a motorized vehicle. More than half had no formal education. Just over one-third of these IDPs had six or more children.

**Table 1:** Sociodemographic characteristics of married men in need of contraception in Afghanistan, 2017 (*n* = 885)

Characteristic	IDP (*n* = 265) n (%)	Total (*n* = 885) n (%)
Country of birth		
Afghanistan	251 (94.7)	849 (95.9)
Outside Afghanistan (Pakistan & Iran)	14 (5.3)	36 (4.1)
Urban vs rural location		
Urban	129 (48.7)	354 (40.0)
Rural	136 (51.3)	531 (60.0)
Province of interview		
Balkh	0	94 (10.6)
Bamyan	0	169 (19.1)
Herat	0	36 (4.1)
Kabul	85 (32.1)	109 (12.3)
Kandahar	74 (27.9)	74 (8.4)
Nangarhar	106 (40.0)	210 (23.7)
Takhar	0	193 (21.8)
Age		(*n* = 883)
18–24 years	46 (17.4)	123 (13.9)
25–30 years	69 (26.1)	248 (28.1)
31–40 years	77 (29.2)	286 (32.4)
41–49 years	72 (27.3)	226 (25.6)
Household items owned		
Radio and/or Television	174 (65.7)	654 (73.9)
Motorized vehicle (Motorcycle and/or car)	85 (32.1)	368 (41.6)
Education		
Any formal education	123 (46.4)	530 (59.9)
No education	142 (53.6)	355 (40.1)
Number of living children		(*n* = 884)
0–1 children	55 (20.8)	196 (22.2)
2–3 children	61 (23.0)	225 (25.4)
4–5 children	59 (22.3)	231 (26.1)
6 or more children	90 (34.0)	232 (26.2)

Slightly more than half of all respondents (52.5%) and almost two-thirds of IDP respondents (65.3%) were classified as not using modern contraception (Table 2). Of these, almost half (48.9%) of all respondents reported not using any FP method with their wives and 3.6% reported using traditional methods (withdrawal only (*n* = 30) or the rhythm/standard days method only (*n* = 1) or withdrawal and rhythm together (*n* = 1), data not shown). Just over one-third of all respondents and less than one-quarter of IDPs reported using modern contraception with their wives. About 9% of the men did not respond to the question on FP use and are included in the descriptive tables (Tables 1, 2 and 3) only.

**Table 2:** Reported family planning use and modern contraceptive methods used with wives among married men in need of contraception in Afghanistan, 2017 (*n* = 885)

	IDP (*n* = 265) n (%)	Total (*n* = 885) n (%)
Reported current FP use with wife		
Using modern contraception	63 (23.8)	338 (38.2)
Not using modern contraception	173 (65.3)	465 (52.5)
No response	29 (10.9)	82 (9.3)
Of those using modern contraception, method(s) used^a^	(*n* = 63)	(*n* = 338)
Oral contraceptives	26 (41.3)	141 (41.7)
Male condoms	27 (42.9)	133 (39.3)
Injectable contraceptives	27 (42.9)	130 (38.5)
Withdrawal^b^	6 (9.5)	18 (5.3)
IUD/ loop	3 (4.8)	11 (3.3)
Female sterilization	0	10 (3.0)
Rhythm method/standard days^b^	4 (6.3)	5 (1.5)
Lactational amenorrhea (LAM)	4 (6.3)	5 (1.5)
Implant	0	3 (0.9)
Emergency Contraception	1 (1.6)	2 (0.6)
Male sterilization	0	1 (0.3)

^a^ Could report multiple methods

^b^ Reported in combination with a modern method

**Table 3:** Reported wife’s household decision-making involvement and IPV attitudes among married men in need of contraception in Afghanistan, 2017 (*n* = 885)

Variable	IDP (*n* = 265) n (%)	Total (*n* = 885) n (%)
Wife’s involvement in household decision-making: wife involved in decision, by decision type		
How income is spent	105 (39.6)	560 (63.3)
Making major household purchases	124 (46.8)	600 (67.8)
Health care for family	139 (52.5)	637 (72.0)
Wife’s involvement in household decision-making: combined variable		
Wife not involved in decisions	96 (36.2)	179 (20.2)
Wife involved in some decisions	89 (33.6)	216 (24.4)
Wife involved in all decisions	80 (30.2)	490 (55.4)
Attitudes towards IPV: Agrees that a husband is justified in hitting or beating his wife if:		
She goes out without telling him	(*n* = 263)	(*n* = 871)
	116 (44.1)	275 (31.6)
She neglects the children		(*n* = 875)
	55 (20.8)	152 (17.4)
She argues with him	(*n* = 260)	(*n* = 867)
	59 (22.7)	167 (19.3)
She burns the food	(*n* = 262)	(*n* = 872)
	32 (12.2)	80 (9.2)
She refuses to have sex with him	(*n* = 245)	(*n* = 801)
	51 (20.8)	116 (14.5)
Attitudes towards IPV: combined variable	(*n* = 253)	(*n* = 831)
IPV justified in ≥1 circumstance	145 (57.3)	386 (46.5)
IPV not justified in any circumstance	108 (42.7)	445 (53.5)

The most commonly used methods were oral contraceptives, condoms and injectable contraceptives. A few respondents reported using a modern method in combination with withdrawal (5.3%) or rhythm (1.5%). Overall, reported withdrawal use, in combination or alone, was somewhat common and long-acting and permanent methods were relatively rarely reported. Just over one-quarter of respondents reported using more than one method with their wives.

Regarding household decision-making, about two-thirds of all respondents and fewer than half of IDPs said their wives were involved in decisions around how income is spent and making major household purchases (Table 3). Reported involvement by wives in family healthcare decisions was relatively higher than other decisions. Depending on the type of household decision, only 1–3% of respondents stated that their wife alone usually makes the decision (data not shown). One-fifth of all men and more than one-third of IDPs said their wives were not involved in any household decisions, about one quarter of all men and one-third of IDPs said they were involved in some decisions, and just over half of all men and less than one-third of IDPs said their wives were involved in all decisions.

Respondents more commonly agreed that IPV was justified if a wife went out without telling her husband and least commonly agreed that IPV was justified if a wife burns the food (Table 3). Overall, some respondents refused to answer or responded “don’t know” for each of the IPV questions; this was most common for the question about whether IPV is justified if a wife refuses to have sex (9% of total sample). Almost half of all men (46.5%) and more than half of IDPs (57.3%) reported believing that IPV was justified in at least one presented circumstance.

Table 4 presents the crude odds ratios (OR) and adjusted odds ratios (AOR) for variables of interest with reported modern contraceptive use. In bivariate analyses, wives’ involvement in some or all household decisions, reporting that IPV is not justified in any listed circumstance, having any formal education, owning a radio and/or television and owning a motorized vehicle were significantly positively associated with reported modern contraceptive use. Being older (compared to being 18–24 years old) and having more children (compared to having no or one child) were also significantly positively associated with modern contraceptive use. Men who were IDPs were significantly less likely to report using modern contraception. Compared to men in Kabul, men from Bamyan and Takhar were significantly more likely to report using modern contraception, while men from Nangarhar were significantly less likely to report using contraception.

**Table 4:** Factors associated with modern contraceptive use among married men in need of contraception in Afghanistan, 2017: Bivariate and multivariable logistic regression

Variable	Crude OR (95% Confidence Intervals) *n* = 803	*p*-value	Adjusted OR (95% Confidence Intervals) *n* = 760	*p*-value
IDP status				
Non-IDP	Ref		Ref	
IDP	0.39 (0.28, 0.54)	< 0.001	1.14 (0.59, 2.19)	0.700
Urban vs rural location				
Rural	Ref		Ref	
Urban	0.85 (0.64, 1.13)	0.267	0.97 (0.62, 1.53)	0.901
Wife’s involvement in household decision-making				
Wife not involved in decisions	Ref		Ref	
Wife involved in some decisions	2.01 (1.24, 3.26)	0.005	1.73 (0.99, 3.03)	0.057
Wife involved in all decisions	4.16 (2.72, 6.37)	< 0.001	2.57 (1.51, 4.37)	< 0.001
Attitudes towards IPV				
IPV justified in ≥1 circumstance	Ref		Ref	
IPV not justified in any circumstance	1.80 (1.34, 2.41)	< 0.001	0.98 (0.67, 1.42)	0.906
Province of interview				
Kabul	Ref		Ref	
Balkh	0.61 (0.33, 1.14)	0.121	0.51 (0.20, 1.27)	0.147
Bamyan	2.14 (1.26, 3.66)	0.005	2.51 (1.03, 6.08)	0.042
Herat	1.06 (0.48, 2.35)	0.880	1.19 (0.44, 3.25)	0.730
Kandahar	0.65 (0.34, 1.26)	0.204	0.71 (0.32, 1.56)	0.392
Nangarhar	0.34 (0.19, 0.59)	< 0.001	0.30 (0.13, 0.66)	0.003
Takhar	3.99 (2.37, 6.72)	< 0.001	3.07 (1.22, 7.69)	0.017
Age	(*n* = 802)			
18–24 years	Ref		Ref	
25–30 years	1.82 (1.13, 2.95)	0.015	0.98 (0.54, 1.79)	0.952
31–40 years	2.26 (1.41, 3.62)	0.001	0.70 (0.35, 1.38)	0.301
41–49 years	1.74 (1.06, 2.85)	0.029	0.29 (0.13, 0.65)	0.002
Owns radio and/or television				
Does not own radio/television	Ref		Ref	
Owns a radio/television	2.14 (1.52, 3.01)	< 0.001	1.69 (1.10, 2.59)	0.016
Owns motorized vehicle				
Does not own motorcycle and/or car	Ref		Ref	
Owns a motorcycle and/or car	2.13 (1.60, 2.84)	< 0.001	1.39 (0.95, 2.04)	0.086
Education level				
No education	Ref		Ref	
Any formal education	1.70 (1.27, 2.28)	< 0.001	1.42 (0.97, 2.08)	0.073
Number of living children	(*n* = 802)			
0–1 children	Ref		Ref	
2–3 children	1.89 (1.24, 2.88)	0.003	2.56 (1.49, 4.40)	< 0.001
4–5 children	2.65 (1.74, 4.05)	< 0.001	4.94 (2.65, 9.21)	< 0.001
6 or more children	1.65 (1.08, 2.54)	0.022	5.75 (2.84, 11.66)	< 0.001

In the multivariable model, wives’ involvement in decision-making was independently significantly associated with reported modern contraceptive use with degree of association strengthening based on level of decision-making involvement (Table 4). By contrast, attitudes supporting IPV and IDP status did not remain significant in the multivariable model. In the multivariable model men in the oldest age group (41–49 years) were significantly less likely to report using contraception than men in the youngest group (18–24 years). No variables with tolerance< 0.10 or condition indexes> 30 were detected in the multivariable model. Also, no significant interactions were detected between IDP status and IPV attitudes and household decision-making and were therefore not included in the final model.

## Discussion

This study contributes novel information about married couples’ modern contraceptive use from the perspective of married men in Afghanistan. Men in general, and IDPs in particular, are often not included in reproductive health research, despite being critical populations to engage for reproductive health care decisions and access. We found relatively low rates of reported modern contraceptive use among this sample of married men and IDPs with identified contraceptive need. Notably, we found a positive independent association between reported participation of wives in household decision-making with reported modern contraceptive use but did not find that attitudes towards IPV were independently associated with reported modern contraceptive use. These findings have important implications for FP programs in Afghanistan.

We found that greater reported involvement in household decision-making by wives was significantly associated with reported modern contraceptive use, consistent with findings from studies, primarily among women, in other settings [21–28]. Slightly more than two-thirds of all men and just under half of IDPs in our study reported that their wives were involved in decisions on major household purchases specifically; this was substantially higher than national estimates where only one-third of married men reported that their wives participate in this decision [3]. A lack of communication about major household purchases may also indicate a lack of spousal communication generally, and specifically about FP. Therefore, engaging men around spousal communication about family size and contraceptive use may be a viable entry point for motivating male support for and uptake of contraception. Spousal communication about FP has been associated with higher contraceptive use in other Asian countries including Nepal and Bangladesh [32, 33]. Fostering spousal communication about FP in various ways has successfully resulted in increasing contraceptive use in other countries [34–36]. For example, using trained male healthcare workers to teach men and couples respectful communication skills about contraception resulted in increased contraceptive communication and contraceptive use among young married couples in rural India [34].

We found that only 38% of married men and 24% of IDPs determined to have a need for contraception reported using modern contraception with their wives. Additionally, withdrawal, a solely male-controlled method, was more commonly reported by men who also reported lower levels of wives’ involvement in household decision-making. This finding may reflect that men who do not involve their wives in decision-making are also more likely to control the couple’s FP use. Use of withdrawal may also be related to misperceptions about modern methods, as men and women felt that only withdrawal and LAM were safe methods in a qualitative study from Afghanistan [15]. Dispelling misconceptions about modern contraception and engaging men both as FP clients and supportive partners could increase joint decision-making, leading to couples selecting more effective, modern methods.

Attitudes justifying IPV have been shown to be correlated with IPV perpetration and are prevalent in settings where men and women have unequal power [3, 31, 37]. According to the Afghanistan DHS, 56% of ever-married women reported ever experiencing IPV (physical, sexual, or emotional) and 52% reported experiencing IPV in the past 12 months [3]. The Afghanistan DHS also found high rates of men’s acceptance of IPV: 72% of men reported that IPV was justified in at least one of the five listed circumstances [3]. In our bivariate analysis, men who reported that IPV was not justified in any stated scenario were nearly twice as likely to report using modern contraception with their wives than those who felt that IPV was justified in at least one circumstance. However, in the multivariable model, IPV attitudes were not significantly associated with modern contraceptive use when controlling for IDP status, rural versus urban residence, age, education, number of children, province, and owning a radio/television or motorized vehicle. Few studies have looked at associations between men’s IPV attitudes and contraceptive use. In a study in Tanzania, wives’ non-acceptance of IPV was positively associated with reported contraceptive use but husbands’ IPV attitudes were not significantly associated with contraceptive use [26]. In an analysis of 1998 DHS data from Turkey, men expressing more gender-equitable attitudes (which included not justifying IPV) were more likely to report contraceptive use [38]. Though IPV attitudes were not independently associated with reported contraceptive use in our study, that nearly half of married male respondents and more than half of IDPs felt that IPV is justified in at least one circumstance is concerning within a context of high IPV prevalence reported by married women [3]. Therefore, our findings support expanding efforts to address and transform attitudes towards IPV among men, women and key influencers.

IDP status was not significantly associated with lower reported modern contraceptive use, which was surprising as studies in other fragile settings document challenges to accessing FP for IDPs [20]. Additionally, though other studies in Afghanistan have documented higher rates of contraceptive use in urban areas, urban versus rural residence was not significantly associated with reported contraceptive use [3, 39, 40]. This observation may be partially explained by the fact that the respondents recruited in urban areas were largely IDPs, who are typically displaced from rural areas, and ANSF groups, who come to urban areas for work from all areas of Afghanistan. Compared to the full sample, fewer IDPs reported that their wives were involved in household decisions and more IDPs reported attitudes which condoned IPV in at least one circumstance. IDP respondents’ attitudes and contraceptive practices are likely influenced by the social and gender norms from the rural communities they were displaced from. Though IDP status was not independently associated with lower odds of modern contraceptive use, reported contraceptive use was quite low and may warrant demand generation and engagement tailored to the needs of this group.

Similar to other studies in Afghanistan, we found that province of interview was significantly associated with reported modern contraceptive use [3, 39]. This finding is consistent with DHS data, which show large variations in modern CPR across Afghanistan and among our study provinces, ranging from 13% in Balkh and Nangarhar to 58% in Herat among married women [3]. The DHS also found large variations between our study provinces in IPV attitudes (e.g. 35% of men in Takhar reported that IPV in at least one circumstance was justified compared to 83–84% of men in Kandahar, Nangarhar and Herat) and wives’ involvement in household decisions (e.g. 14% of married women in Kandahar reported being involved in making major household purchases compared to 80% in Takhar) [3]. These variations suggest different underlying social and gender norms and differences in fertility preferences and attitudes towards modern contraception in different areas of Afghanistan.

We viewed owning a radio and/or television as an indicator of potential mass media exposure and socioeconomic status. Ownership of either of these items was positively associated with contraceptive use in our study, similar to findings from a study of women’s contraceptive use in Afghanistan [39] and consistent with radio and television being the most commonly reported channels for FP messaging among married men [3]. Our findings support reaching men with messages related to modern contraception through mass media. Furthermore, owning a motorized vehicle may indicate that household wealth and mobility may increase service accessibility. However, we did not find strong evidence that men who own a motorized vehicle were more likely to report using modern contraception. Given that fewer IDPs owned a radio and/or television or motorized vehicle, additional outreach methods may need to be tested to reach this group.

This study has some limitations that should be considered when interpreting our findings. The data are cross-sectional and therefore cannot establish causality. Further, due to non-probability sampling, our results cannot be considered representative of men overall, or for a specific group (e.g. IDPs), in Afghanistan. It is possible that we did not account for other potential confounding factors in our multivariable model. Generally, reproductive health-related issues are sensitive topics and not easily discussed in Afghanistan [10], particularly in some regions and among some specific populations. In this study, nearly 10% of men did not respond to the questions about FP use and whether IPV was justified if a wife refuses to have sex, which may have influenced our results. Additionally, the parent study generally did not sample from predominantly rural provinces, which may have different contraceptive practices and social and gender norms. Sampling decisions were based on logistical feasibility and access to sufficient numbers of target groups in a time-efficient manner, though some predominantly rural provinces (e.g. Takhar, Bamyan) and districts (e.g. Kama) were included. It is possible that FP use may have been over- or under-reported by men based on perceived socially desirable response; similarly, this may have been the case with justification of IPV or their wife’s participation in household decision-making. Additionally, since we did not corroborate men’s reports of FP use with their wives, it is possible that these women could be using contraception without their husbands’ explicit knowledge [11]. Finally, some people are ambivalent about having more children and fertility desires can change over time, therefore there is potential for misclassification bias in our definition of “in need of contraception”.

## Conclusions

Our findings further support engaging men as FP clients and beneficiaries, supportive partners, and agents of positive change [41–43]. The majority of respondents reported that their wife participated in at least some household decisions, and we found increased odds of reported contraceptive use with greater wives’ involvement in household decisions. These data suggest that engaging men around spousal communication may be a viable entry point for motivating male support for and uptake of contraception.

Despite evidence that male engagement is an important strategy for FP program success, male engagement in FP programming in Afghanistan to date has been limited [9, 16, 44, 45]. More recently, Afghanistan’s MoPH has approved a National Reproductive, Maternal, Newborn, Child and Adolescent Health Strategy 2017–2021 [2] and a Birth Spacing/Family Planning Costed Implementation Plan (2018–2022) [45] that mention male engagement in FP. However, to realize the impact of male engagement on modern contraceptive use in Afghanistan, programming needs to be taken to scale and additional models developed. Multiple strategies are likely necessary to reach IDPs.

Gender-transformative approaches, which include challenging existing unequal gender norms and promoting gender equitable attitudes and roles, have successfully increased modern contraceptive use and gender-equitable decision-making, and decreased IPV in other countries such as India and Rwanda [34, 46]. Using adapted gender-transformative models from other countries which incorporate the male involvement model may help the government of Afghanistan achieve their FP goals.

## Data Availability

The dataset from which this analysis was derived is available through Harvard’s Dataverse at the following link: 10.7910/DVN/QULMNN

## Abbreviations

ANSF: Afghan National Security Forces
AOR: Adjusted odds ratio
ATR: Assess, Transform & Reach Consulting
CI: Confidence interval
CPR: Contraceptive prevalence rate
DHS: Demographic and Health Survey
FP: Family planning
FP/RMNCH: Family planning and reproductive, maternal, newborn and child health
HEMAYAT: Helping Mothers & Children Thrive in Afghanistan project
IDP: Internally displaced persons
IPV: Intimate partner violence
IUD: Intrauterine device
LAM: Lactational amenorrhea
MoPH: Ministry of Public Health
OR: Odds ratio
RMNCH: Reproductive, maternal, newborn and child health
USAID: United States Agency for International Development
